# Utility of hemoglobin A1c in detecting risk of type 2 diabetes: comparison of hemoglobin A1c with other biomarkers

**DOI:** 10.3164/jcbn.19-16

**Published:** 2019-05-10

**Authors:** Aya Umeno, Yasukazu Yoshida

**Affiliations:** 1 Health Research Institute, National Institute of Advanced Industrial Science and Technology, 2217-14 Hayashi-cho, Takamatsu, Kagawa 761-0395, Japan

**Keywords:** hemoglobin A1c, risk of type 2 diabetes, biomarkers, prediction algorithm, glucose tolerance

## Abstract

We have previously reported that the risk of type 2 diabetes, early impaired glucose tolerance, and insulin resistance can be predicted using fasting levels of adiponectin, leptin, and insulin. Here, we aimed to evaluate the utility of hemoglobin A1c in detecting the risk of type 2 diabetes compared with other well-known biomarkers. We randomly enrolled 207 volunteers with no history of diseases, who underwent 75-g oral glucose tolerance tests and were stratified into normal, borderline, abnormal, or diabetic groups. Eighty-one participants with normal baseline levels of hemoglobin A1c (<6.0%) were included in the normal groups of both glucose tolerance and insulin resistance. Hemoglobin A1c was significantly correlated with the plasma glucose and insulin resistance index. Leptin, adiponectin, glycoalbumin, and body mass index also were correlated well with plasma glucose levels and insulin resistance index. Normal hemoglobin A1c levels with abnormal glucose tolerance and insulin resistance were noted in 85 and 67 participants, respectively. Hemoglobin A1c did not strengthen the prediction algorithm of diabetes, determined by our proposed biomarkers, leptin, adiponectin, and insulin. In conclusion, hemoglobin A1c is a surrogate biomarker for risk of diabetes, with inadequate predictive value, and should be used in combination with other biomarkers.

## Introduction

Early detection and treatment of diabetes is important as it can delay or prevent serious complications associated with type 2 diabetes (T2D) such as blindness, amputation, and renal disease. The Japan Diabetic Society classifies individuals with fasting plasma glucose (FPG) levels of 100–109 mg/dl as having “high-normal” (HN) glucose metabolism. Individuals with an FPG of >110 mg/dl and hemoglobin (Hb)A1c of >6.0% are recommended to undergo 75-g oral glucose tolerance test (OGTT) to determine whether they are normal, borderline diabetic, or diabetic.^(1)^ Impaired fasting glucose (IFG) and impaired glucose tolerance (IGT) are pre-diabetic states that represent intermediate phases during the transition from normal glucose tolerance to diabetes.^(2)^ IFG and IGT are defined based on the OGTT results.^(3)^

HbA1c is an important and useful biomarker for detecting impaired glucose tolerance and for diabetes treatment. However, there are several limitations in the use of HbA1c for diagnosing T2D. In particular, the level of HbA1c varies depending on the turnover rate of erythrocytes and the levels of glycaemia. Pregnancy, renal anemia, chronic and hemolytic anemia, acute blood loss, liver disease, dialysis, and chronic malaria cause low HbA1c levels.^(4)^ Vitamins C and E have also been reported to lower HbA1c levels by inhibiting glycation.^(5)^ Meanwhile, HbA1c levels may be higher in individuals with a longer erythrocyte life span, such as those with vitamin B12 or folate deficiency.^(6)^

FPG and HbA1c are inadequate for early detection of both IGT and insulin resistance. Accordingly, several studies have been performed to identify accurate biomarkers for detecting these conditions. Oxidative stress is a common pathogenic factor that is hypothesized to lead to insulin resistance, β-cell dysfunction, IGT, and IFG. We recently proposed 10- and 12-(*Z*,*E*)-hydroxyoctadecadienoic acid (HODE) as a possible biomarker for diabetes. HODE, which is yielded by singlet oxygen oxidations,^(7,8)^ is strongly correlated with insulin resistance. Considerable research has been focused on the products of lipid peroxidation as lipids are susceptible to *in vivo* oxidation. Various lipid products have been evaluated. Among them, evaluation of F_2_-isoprostanes consisting of a series of chemically stable prostaglandin F_2_-like compounds is considered a gold standard for the evaluation of oxidative stress *in vivo*.^(9,10)^ The formation of 9-hydroxy linoleic acids in the erythrocyte membranes of patients with diabetes^(11)^ and hydroxy fatty acid in patients with atherosclerosis^(12)^ has been studied previously.

Other biomarkers have also been evaluated and used for the prediction of diabetes, including adiponectin,^(5)^ leptin,^(13,14)^ glycoalbumin,^(15)^ and retinol binding protein 4 (RBP4).^(16,17)^ Fat accumulation in the human body releases several adipokines from adipocytes, and some of these adipokines are known to aggravate insulin resistance, cause metabolic syndrome, and promote T2D.

Adiponectin and leptin are the most commonly used biomarkers in clinical practice and are also used in diabetes screening. Glycated albumin is shown as the percentage of serum glycated albumin (same as HbA1c) in the total serum albumin and is considered to be a better indicator of short-term glycemic control compared with HbA1c as albumin is reduced by 50% within 2–3 weeks.^(15)^ RBP4 is an adipocyte-derived factor and acts on muscle and/or liver by either retinol-dependent or independent mechanism. RBP4 is primarily produced in the liver and has recently been reported to be involved in the early phases of adiposity and insulin resistance.^(18)^

In this study, we aimed to evaluate the usefulness of HbA1c for detecting the risk of T2D compared with other well-known biomarkers. Further, we intended to provide more surrogate combinations of biomarkers to detect T2D at annual health examinations other than HbA1c and FPG.

## Materials and Methods

### Materials

The materials used were of the highest grade that was commercially available.

### Participants and sample processing

We randomly enrolled healthy volunteers who had no history of any diseases. They underwent a 120-min 75-g OGTT after more than 10 h fasting, with blood collected every 30 min in tubes containing ethylenediaminetetraacetic acid disodium salt. Plasma and erythrocytes were separated immediately after collection via centrifugation at 1,500 × *g* for 10 min at 4°C. Subsequently, plasma was frozen and stored at −80°C until analysis. This study was approved by the institutional review boards of the National Institute of Advanced Industrial Science and Technology, Olive Takamatsu Medical Clinic, and Tokushima University, and was performed in accordance with the ethical standards established in the 1964 Declaration of Helsinki and its later amendments. All participants provided written informed consent after the purpose of this study was thoroughly explained.

### Biomarkers

HbA1c, glucose, insulin, leptin, adiponectin, RBP4, glycoalbumin, and hs-CRP were measured using commercially available ELISA kits {HbA1c: RAPIDIA Auto HbA1c-L [Fujirebio Inc. (Tokyo, Japan)]; glucose: Cica liquid GLU J [KANTO CHEMICAL Co., Inc. (Tokyo, Japan)]; insulin: Lumipulse Presto Insulin (Fujirebio Inc.); leptin: Human leptin RIA kit [Millipore Co., Inc. (Tokyo, Japan)]; adiponectin: CircuLexTM Human adiponectin ELISA Kit CY-8050 [MBL Co., Ltd. (Nagano, Japan)]; RBP4: CircuLex Human RBP4 ELISA Kit (MBL Co., Ltd.); glycoalbumin: Lucia GA-L [Asahi KASEI Pharma Co., Inc. (Tokyo, Japan)]; and hs-CRP: CircuLexTM Human HS-CRP ELISA Kit CY-8071 (MBL Co., Ltd.)}. Oxidative stress markers were measured according to our previous reports.^(7,8,19)^ In this study, normal HbA1c was defined at 4.6 to 5.5%, while HbA1c levels >6.5% were considered to indicate diabetes. The HbA1c levels of 5.5–6.5% are considered to be borderline, and the diagnosis and treatment by physicians vary depending on the the protocol followed in the clinical setting in different countries.

### Non-invasive indices

We measured several non-invasive indices including height, weight, waist circumference, diastolic blood pressure, systolic blood pressure, pulse-wave velocity, and abdominal fat distribution. The last two have been linked to early stage diabetes.^(20)^ The branchial ankle pulse wave velocity of each subject was measured using an automatic oscillometer (PWV/ABI, BP-203RPE; Omron Collin, Tokyo, Japan) with subjects in the supine position after 5 min of bed rest. Abdominal fat distribution was determined by computed tomography with the subjects in the same position. Subcutaneous and intra-abdominal fat were measured at the level of the umbilicus by computed tomography.

### Statistical methods

One-factor repeated measures design analysis of variance was used to examine the main effect of elapsed time from glucose injection on each index. Significant effects were then assessed using Tukey’s honestly significant difference multiple comparisons. Correlations were also analyzed using Pearson test. Statistical analyses were performed using IBM SPSS software version 21.0 (IBM Corp., New York, NY). A *p* value of <0.05 was considered significant. Data were expressed as mean ± SD.

## Results

A total of 207 participants (male, 179; female, 28) were enrolled. Initially, there was an equal number of male and female volunteers. However, we found that the biomarkers we measured were influenced by physiological variations specific to women such as menstruation. Thus, we also analyzed data collected from men thereafter. In the overall population, the age ranged from 26 to 63 years (46 ± 7 years); it was 26 to 63 years (47 ± 6 years) in men and 26 to 62 years (39 ± 8 years) in women. We showed the overall results and those obtained by sex.

**Fig. 1:**
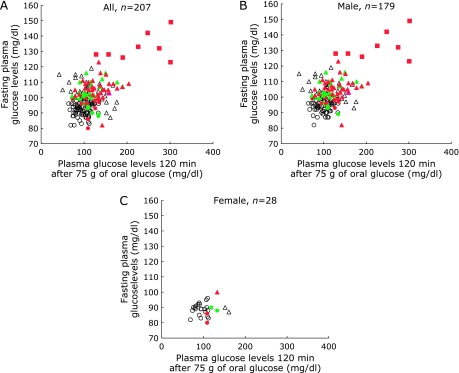
Classification of glucose tolerance and insulin resistance using the oral glucose tolerance test. Circle, group N (normal); triangle, group HN + IGT (“high-normal” and impaired glucose tolerance); square, group D (diabetic). Open, normal insulin resistance; green, borderline insulin resistance; red, abnormal insulin resistance. Insulin resistance was determined by homeostasis model assessment of insulin resistance (HOMA-IR) and Matsuda Index 3.

Figure 1 shows the OGTT results for all (A), male (B), and female (C) participants. Glucose tolerance and insulin resistance are also indicated using marks and color, respectively. Both IFG and IGT were determined according to the levels of FPG and 2 h OGTT results. We found that women had better glucose levels than did men (Fig. 1B and C), but the reason was unknown. We primarily focused on HbA1c levels <6.5% to determine which participants in the abnormal groups with glucose tolerance had normal HbA1c levels. Surprisingly, in our cohort, the levels of HbA1c of all participants in the high normal, IFG, and/or IGT groups were under 6.5%. Notably, more than 50% of the participants in these groups showed the levels of HbA1c <6.0%. We then lowered the HbA1c cut-off from 6.5% to 6.0% because in Japan, physicians recommend further evaluation when the HbA1c level is beyond 6.0%. Table 1 shows the HbA1c levels according to glucose categories of the patients. As shown, almost all participants (98/99 participants) with normal glucose tolerance had an HbA1c of <6.0%, irrespective of the insulin resistance condition. Additionally, the levels of HbA1c in the participants with diabetes were higher than 6.0%, except in one participant whose level was 5.9. Surprisingly, 84% (84/100) of the subjects in the HN, IFG, and/or IGT groups had HbA1c levels <6.0%. In Japan, only FPG and HbA1c levels are measured in annual health examinations. Thus, HN and IGT participants whose HbA1c levels were <6.0% were not recommended for further examination. In this study, 62 participants had FPG levels below 110 and had an insulin resistance index of borderline or abnormal. Additionally, the number of male participants with borderline and abnormal glucose tolerance and insulin resistance were higher than that of females.

We then further analysed the participants with borderline and insulin resistance. As shown in Fig. 1 and Table 1, 67 participants who had HbA1c levels <6.0% showed borderline and abnormal insulin resistance, irrespective of evaluation methods [i.e., Homeostasis Model Assessment of Insulin Resistance (HOMA-IR) and Matsuda Index 3 (MI3)].

We also investigated the biomarkers for predicting glucose tolerance and insulin resistance, including oxidative stress markers and non-invasive indices such as body mass index (BMI). The significances of their correlation are summarized in Table 2. In the overall study cohort, HbA1c was significantly correlated with fasting plasma levels of insulin, MI3, and HOMA-IR, but it did not strongly correlate with high-sensitivity C-reactive protein (hs-CRP), leptin, adiponectin, or OGTT (1 h) insulin. Meanwhile, BMI, leptin, and adiponectin showed a significant correlation with all levels of glucose and insulin during OGTT, and their correlation was stronger than that of HbA1c. By contrast, glycoalbumin and RBP4 did not show a good correlation with glucose and insulin levels. This finding was almost similar to that obtained from male participants. By contrast, the correlation was not as strong in female participants, which may be due because 75% of them had no impaired glucose tolerance or insulin resistance as shown in Table 1. Meanwhile, the levels of hs-CRP were significantly correlated with insulin levels during OGTT, but the reason for this finding was unknown. Additionally, leptin and BMI correlated fairly well with insulin levels during OGTT among the female participants.

## Discussion

Previous studies reported that the combination of the fasting levels of leptin, adiponectin, and insulin predicted the onset of T2D^(8,21)^ without OGTT, and their accuracy was improved by the addition of 10, 12-(*Z*,*E*)-HODE. In this study, we evaluated the usefulness of HbA1c as a biomarker for the early identification of the risk for T2D. Our findings clearly demonstrated that HbA1c as well as leptin, adiponectin, and insulin were strongly related to both glucose tolerance and insulin resistance (Table 2). However, HbA1c solely could not predict either glucose tolerance or insulin resistance perfectly. For example, 85 participants (41% of the total) were showing abnormal glucose tolerance but their HbA1c levels were normal (<6.0%). Further, 67 participants (32%) whose HbA1c levels were normal exerted insulin resistance determined by OGTT. In other words, around half of the participants (102/207) were normal levels of HbA1c but showed abnormality in either glucose tolerance or insulin resistance. Accordingly, the clinical value of HbA1c for the prediction was not exclusively assessed in this study. This is reasonable because HbA1c reflects conditions for 3 to 4 months, and the level varies during small range. Moreover, it should be noted that combining HbA1c with leptin, adiponectin, and insulin could not provide better results on the statistical analysis.

Assessing *in vivo* insulin secretion is complex because it is influenced by multiple factors including systemic insulin sensitivity, hepatic insulin extraction, plasma-free fatty acids, and glucolipid toxicity.^(5)^ Insulin resistance is determined via HOMA-IR using the formula FPG × fasting insulin level/405, and the Japan Diabetes Society^(2)^ defines a normal level as <1.6 and insulin resistance as >2.5. Insulin resistance can also be determined via the MI, which is calculated based on plasma glucose and insulin levels determined in OGTT. MI3 points are measured as 10,000/([FPG × fasting plasma insulin level] × [mean OGTT glucose level × mean OGTT insulin level])^1/2^ at 0, 60, and 120 min after the start of the OGTT, where a normal level is >3.^(22)^ Although glucose tolerance and insulin homeostasis are important factors for evaluating the risk of diabetes, few people in Japan undergo OGTT because it is a time-consuming, costly, and only an optional test. Furthermore, when OGTT is performed, glucose levels 120 min after the test are occasionally measured without insulin data, resulting in a lack of information on insulin homeostasis. For this reason, it is very important to propose fasting levels of biomarkers, which quantitatively show the states of glucose tolerance and insulin resistance.

The pathogenesis of T2D is well documented.^(23)^ Based on the mechanism, many biomarkers for the prediction of T2D are proposed.^(24,25)^ Among them, HbA1c is a gold standard for the evaluation of glucose tolerance since it reflects the glucose levels directly in the blood, although it is not a sensitive marker. Insulin resistance is characterized by increased glucose uptake resulting from decreased peripheral muscle glucose uptake. Increased lipolysis and plasma free fatty acid levels stimulate glucose output, reduce peripheral glucose utilization, and impair β-cell function. Preventing β-cell dysfunction is critical for preventing T2D development and progression. Compensatory insulin secretion by pancreatic β-cells may initially maintain normal plasma glucose levels, but β-cell function is already abnormal at this stage; concomitantly, there is inappropriate glucagon release from pancreatic β-cells. Glucagon-like peptide-1 and glucose-dependent insulinotropic polypeptide (incretin) enhance β-cell insulin secretion and are therefore therapeutic targets for T2D treatment.^(19)^ The effects of ominous octet (increased lipolysis, glucose reabsorption, hepatic glucose production in the liver, and glucagon secretion; decreased glucose uptake, insulin secretion, and incretin levels; and neurotransmitter dysfunction) lead to hyperglycemia in T2D. Accordingly, these two peptides are considered appropriate biomarkers for detecting the onset of diabetes. However, to our knowledge, there are no effective antibodies against both peptides, resulting in the lack of quantitative data.

Continuous follow-up of the health status is important to prevent lifestyle-related diseases including diabetes. In this regard, surrogate biomarkers are needed. As stated previously, FPG and HbA1c should both be assessed in annual health check-ups as using only one is inadequate for predicting glucose tolerance and insulin resistance. However, it may drop the borderline patients even if both biomarkers are used, as shown in this study. We have previously proposed the multiple linear regression model by using adiponectin, leptin, and insulin for the detection of diabetic risk.^(8,19)^

In conclusion, HbA1c was correlated significantly with plasma glucose levels during OGTT and with MI3 or HOMA-IR. However, glucose tolerance and insulin resistance cannot be accurately predicted using only FPG and HbA1c. We propose that these markers should be used in combination with adiponectin, leptin, and insulin for the early detection of the risk of diabetes.

## Author Contributions

AU, experiment, analysis, and writing paper; YY, planning experiment and writing paper.

## Figures and Tables

**Table 1:** Relatonship between the levels of HbA1c and glucose tolerance

*n* = 207 (all)				
Glucose tolerance	Insulin resistance	Nomber of subjects	%	Number of HbA1c <6.0%
Normal (〇)	Normal (Black)	82	39.6	81
Normal (〇)	Border (Green)	7	3.4	7
Normal (〇)	Abnormal (Red)	10	4.8	10
High-normal, IFG and/or IGT (△)	Normal (Black)	43	20.8	35
High-normal, IFG and/or IGT (△)	Border (Green)	20	9.7	15
High-normal, IFG and/or IGT (△)	Abnormal (Red)	37	17.9	34
Diabetec (□)	Abnormal (Red)	8	3.9	1

Total		207	100	183

*n* = 179 (Man)				
Glucose tolerance	Insulin resistance	Nomber of subjects	%	Number of HbA1c <6.0%
Normal (〇)	Normal (Black)	61	34.1	60
Normal (〇)	Border (Green)	5	2.8	5
Normal (〇)	Abnormal (Red)	8	4.5	8
High-normal, IFG and/or IGT (△)	Normal (Black)	41	22.9	33
High-normal, IFG and/or IGT (△)	Border (Green)	20	11.2	15
High-normal, IFG and/or IGT (△)	Abnormal (Red)	36	20.1	33
Diabetec (□)	Abnormal (Red)	8	4.5	2

Total		179	100	156

*n* = 28 (Female)				
Glucose tolerance	Insulin resistance	Nomber ofsubjects	%	Number of HbA1c <6.0%
Normal (〇)	Normal (Black)	21	75	21
Normal (〇)	Border (Green)	2	7.1	2
Normal (〇)	Abnormal (Red)	2	7.1	2
High-normal, IFG and/or IGT (△)	Normal (Black)	2	7.1	2
High-normal, IFG and/or IGT (△)	Border (Green)	0	0	0
High-normal, IFG and/or IGT (△)	Abnormal (Red)	1	3.6	1
Diabetec (□)	Abnormal (Red)	0	0	0

Total		28	100	28

a: % are the % of total participants classified according to glucose tolerance and insulin resistance. The mark and color in parantheses are glucose tolerance and insulin resistance, respectively.

**Table 2:** Correlation between the glucose and inslurin resistance during fasting and OGTT and fasting levels of multi-biomaekers

*n* = 207 (all)								*n* = 152 (male only)	
	HbA1c	hsCRP	Leptin	Adiponectin	BMI	Ages		Glycoalbumin	RBP4
Glu 0 min	*p* = 0.000 **	*p* = 0.357	*p* = 0.019 *	*p* = 0.006^ ##^	*p* = 0.000 **	*p* = 0.000 **		—	—
Glu 60 min	*p* = 0.000 **	*p* = 0.237	*p* = 0.003 **	*p* = 0.019 ^#^	*p* = 0.000 **	*p* = 0.002 **		—	—
Glu 120 min	*p* = 0.000 **	*p* = 0.011 *	*p* = 0.000 *	*p* = 0.001 ^##^	*p* = 0.000 **	*p* = 0.265		—	—
Insulin 0 min	*p* = 0.000 **	*p* = 0.342	*p* = 0.000 **	*p* = 0.000 ^##^	*p* = 0.000 **	*p* = 0.289		—	—
Insulin 60 min	*p* = 0.083	*p* = 0.412	*p* = 0.000 **	*p* = 0.002 ^##^	*p* = 0.000 **	*p* = 0.473		—	—
Insulin 120 min	*p* = 0.016 *	*p* = 0.058	*p* = 0.000 **	*p* = 0.000 ^##^	*p* = 0.000 **	*p* = 0.656		—	—
MI3	*p* = 0.000 ^##^	*p* = 0.051	*p* = 0.000 ^##^	*p* = 0.000 **	*p* = 0.000 ^##^	*p* = 0.046 ^#^		—	—
HOMA-IR	*p* = 0.000 **	*p* = 0.367	*p* = 0.000 **	*p* = 0.000 ^##^	*p* = 0.000 **	*p* = 0.120		—	—
HbA1c	—	*p* = 0.712	*p* = 0.063	*p* = 0.363	*p* = 0.024 *	*p* = 0.000 **		—	—

*n* = 179 (male)								*n* = 152 (male only)	
	HbA1c	hsCRP	Leptin	Adiponectin	BMI	Ages		Glycoalbumin	RBP4
Glu 0 min	*p* = 0.000 **	*p* = 0.130	*p* = 0.000 **	*p* = 0.015 ^#^	*p* = 0.001 **	*p* = 0.011 *		*p* = 0.060	*p* = 0.103
Glu 60 min	*p* = 0.000 **	*p* = 0.181	*p* = 0.000 **	*p* = 0.056	*p* = 0.000 **	*p* = 0.175		*p* = 0.012 *	*p* = 0.851
Glu 120 min	*p* = 0.000 **	*p* = 0.012 *	*p* = 0.000 **	*p* = 0.003 ^##^	*p* = 0.000 **	*p* = 0.039		*p* = 0.043 *	*p* = 0.310
Insulin 0 min	*p* = 0.001 **	*p* = 0.569	*p* = 0.000 **	*p* = 0.001 ^##^	*p* = 0.000 **	*p* = 0.635		*p* = 0.012 ^#^	*p* = 0.074
Insulin 60 min	*p* = 0.253	*p* = 0.600	*p* = 0.000 **	*p* = 0.008 ^##^	*p* = 0.000 **	*p* = 0.838		*p* = 0.000 ^##^	*p* = 0.535
Insulin 120 min	*p* = 0.048 *	*p* = 0.107	*p* = 0.000 **	*p* = 0.002 ^##^	*p* = 0.000 **	*p* = 0.953		*p* = 0.081	*p* = 0.357
MI3	*p* = 0.002 ^##^	*p* = 0.092	*p* = 0.000 ^##^	*p* = 0.000 **	*p* = 0.000 ^##^	*p* = 0.458		*p* = 0.004 **	*p* = 0.210
HOMA-IR	*p* = 0.000 **	*p* = 0.487	*p* = 0.000 **	*p* = 0.002 ^##^	*p* = 0.000 **	*p* = 0.447		*p* = 0.067	*p* = 0.062
HbA1c	—	*p* = 0.543	*p* = 0.002 **	*p* = 0.576	*p* = 0.045 *	*p* = 0.004 **		*p* = 0.001 **	*p* = 0.790

*n* = 28 (female)									
	HbA1c	hsCRP	Leptin	Adiponectin	BMI	Ages		Glycoalbumin	RBP4
Glu 0 min	*p* = 0.064	*p* = 0.344	*p* = 0.772	*p* = 0.744	*p* = 0.220	*p* = 0.037 *		—	—
Glu 60 min	*p* = 0.469	*p* = 0.424	*p* = 0.680	*p* = 0.480	*p* = 0.670	*p* = 0.214		—	—
Glu 120 min	*p* = 0.938	*p* = 0.420	*p* = 0.325	*p* = 0.480	*p* = 0.220	*p* = 0.913		—	—
Insulin 0 min	*p* = 0.937	*p* = 0.000 **	*p* = 0.000 **	*p* = 0.164	*p* = 0.001 **	*p* = 0.814		—	—
Insulin 60 min	*p* = 0.379	*p* = 0.017 *	*p* = 0.084	*p* = 0.151	*p* = 0.289	*p* = 0.494		—	—
Insulin 120 min	*p* = 0.470	*p* = 0.002 **	*p* = 0.000 **	*p* = 0.127	*p* = 0.009 **	*p* = 0.523		—	—
MI3	*p* = 0.982	*p* = 0.017 ^#^	*p* = 0.001 ^##^	*p* = 0.314	*p* = 0.023^ #^	*p* = 0.830		—	—
HOMA-IR	*p* = 0.783	*p* = 0.000 **	*p* = 0.000 **	*p* = 0.181	*p* = 0.003 **	*p* = 0.958		—	—
HbA1c	—	*p* = 0.801	*p* = 0.911	*p* = 0.956	*p* = 0.677	*p* = 0.254		—	—

The correlation *p* values were calculated by Pearson’s rank correlation test. Red character means no significance. The *,^#^ and **,^##^ correspond to the significance levels at 5% (*p*<0.05) and 1% (*p*<0.01), respectively. The * and ^#^ indicate positive and negative correlations, respectively. Glu 0, 60, 120 min mean the leveles of glucose of 0, 60, 120 min after 75 g of oral glucose, respectively. Insulin 0, 60, 120 min mean the leveles of insulin of 0, 60, 120 min after 75 g of oral glucose, respectively. MI3, Matsuda Index 3; HOMA-IR, homeostasis model assessment of insulin resistance; hsCRP, high-sensitive C-reactive protein.
